# Lp(a) Levels in Relatives of Patients with Acute Coronary Syndrome and Elevated Lp(a): HER(a) Study

**DOI:** 10.3390/jcm13082256

**Published:** 2024-04-12

**Authors:** M. Rosa Fernández-Olmo, Magdalena Carrillo Bailen, Mar Martínez Quesada, Carmen Rus Mansilla, Miriam Martin Toro, Ana López Suarez, Marta Lucas García, Gustavo Cortez Quiroga, Beatriz Calvo Bernal, Samuel Ortiz Cruces, Javier Torres Llergo, Ana García Ruano, Manuel Fernández Anguita, Diego Franco, Alberto Cordero

**Affiliations:** 1 Cardiac Prevention and Rehabilitation Unit Cardiology, University Hospital of Jaén, 23007 Jaén, Spain; m.carrillobailen@outlook.com (M.C.B.); javiertorresllergo@gmail.com (J.T.L.); manueljosefernandezanguita@gmail.com (M.F.A.); 2 Cardiac Rehabilitation Unit Cardiology, Virgen Macarena University Hospital, 41009 Seville, Spain; marquesada@gmail.com (M.M.Q.); martalucas1995@gmail.com (M.L.G.); 3 Alto Guadalquivir Hospital in Andújar, 23740 Jaén, Spain; crusmansilla@gmail.com (C.R.M.); gusacortez@gmail.com (G.C.Q.); 4 Cardiac Rehabilitation Unit Cardiology, University Hospital of Puerto Real, 11510 Cadiz, Spain; mir.martoro@gmail.com (M.M.T.); beacalvo27@gmail.com (B.C.B.); 5 Cardiac Rehabilitation Unit Cardiology, Juan Ramón Jiménez Hospital, 21005 Huelva, Spain; alopezsuarez.als@gmail.com (A.L.S.); samuelortizcruces@hotmail.com (S.O.C.); 6 Clinical Analysis and Genetics Service, University Hospital of Jaén, 23007 Jaén, Spain; anabelengarciaruano@gmail.com; 7 Cardiovascular Research Group, Department of Experimental Biology, University of Jaén, 23071 Jaén, Spain; dfranco@ujaen.es; 8 Cardiology Unit, IMED Elche Hospital, 03203 Alicante, Spain; acorderofort@gmail.com

**Keywords:** lipoprotein(a), cascade diagnosis, ACS, cardiovascular risk

## Abstract

**Background:** Lipoprotein(a) [Lp(a)] is a proatherogenic particle associated with increased cardiovascular risk. It is mainly genetically determined; so, the aim of our study is to evaluate the levels of Lp(a) in the relatives of a prospective cohort of patients who have suffered from an acute coronary syndrome (ACS) with Lp(a) ≥ 50 mg/dL. **Methods**: We conducted a multicenter prospective study, in which consecutive patients who had suffered from an ACS and presented Lp(a) ≥ 50 mg/dL and their first-degree relatives were included. **Results**: We included 413 subjects, of which 56.4% were relatives of the patients. Family history of early ischemic heart disease was present in 57.5%, and only 20.6% were receiving statin treatment. The family cohort was younger (37.5 vs. 59.1 years; *p* < 0.001), and 4% had ischemic heart disease and fewer cardiovascular risk factors. Mean Lp(a) levels were 64.9 mg/dL, 59.4% had levels ≥ 50 mg/dL, and 16.1% had levels ≥ 100 mg/dL. When comparing the patients with respect to their relatives, the mean level of Lp(a) was lower but without significant differences regarding the levels of LDLc, ApoB, and non-HDL. However, relatives with Lp(a) ≥ 50 mg/dL, had values similar to the group of patients with ACS (96.8 vs. 103.8 mg/dL; *p* = 0.18). No differences were found in Lp(a) levels in relatives based on the other lipid parameters. **Conclusions**: Overall, 59.4% of the first-degree relatives of patients who suffered from an ACS with Lp(a) ≥ 50 mg/dL also had elevated levels. Relatives with elevated Lp(a) had similar levels as patients.

## 1. Introduction

Lipoprotein(a) [Lp(a)] is one of the proatherogenic lipoprotein particles associated with increased cardiovascular risk [1]. Its structure is similar to low-density lipoproteins (LDLs) with an ApoB100 and is bound to an apoprotein(a) [Apo(a)] linked by disulfide bonds [2].

In different studies, it has been observed that elevated Lp(a) levels lead to greater cardiovascular risk and increase the probability of thrombotic diseases and aortic stenosis [3]. In a meta-analysis, Larsson et al. observed that when the concentration of Lp(a) is greater than 50 mg/dL, there is a1.36 times higher probability of ischemic coronary events, 1.42 times higher probability of peripheral arterial disease, and 1.74 times higher probability of aortic stenosis [4]. Furthermore, the cardiovascular risk associated with Lp(a) is independent of LDL cholesterol (LDLc) levels, and even in those with optimal levels, there is an increased risk when Lp(a) is elevated [5,6].

Blood levels of Lp(a) are genetically determined in an autosomal dominant fashion [7] and are inversely related to the size of Apo(a), specifically with the number of repeats at the Kringle level [8], and the greater the number of repeats, the greater the size and the lower the concentration of Lp(a). A single accurate measurement is recommended as an efficient method to inform the individual risk associated with Lp(a) [3,7]

In the Heritage study, more than 25% of patients with established cardiovascular disease were found to have Lp(a) levels ≥ 50 mg/dL, with levels being higher in blacks, younger patients, and women [9]. Regarding family members, it is known from population studies that one in five could also have elevated Lp(a) [10]. The aim of our study is to determine the Lp(a) levels of first-degree relatives of patients who have already had an acute coronary syndrome (ACS) and whose Lp(a) levels reached or exceeded 50 mg/dL.

## 2. Methods

We designed an observational, prospective, and multicenter study to assess the distribution of Lp(a) in first-degree relatives, without cardiovascular disease, of patients recently admitted for an ACS and with Lp(a) ≥ 50 mg/dL. The study protocol and the informed consent process were approved by the Ethics Committee of the Biomedical Research Ethics Coordinating Committee of Andalusia. The inclusion period was from February 2022 to February 2023. The sample size was calculated as follows: In published registries [9], it has been observed that approximately 25% of patients with ACS have Lp(a) levels equal to or greater than 50 mg/dL. To achieve an accuracy of 4.25% in the estimation of a proportion using a bilateral normal 95% asymptotic confidence interval, assuming that the proportion is 25% and taking into account that the expected dropout rate is 10%, it would be necessary to recruit more than 300 subjects (patients and relatives) in this study. Calculations were made with ene^®^ 3.0.

### 2.1. Relatives’ Cohort

Once the index case was identified (ACS and Lp(a) ≥ 50 mg/dL), they were informed of the possibility of their first-degree relatives (children, siblings, father, or mother) participating in this study. Subsequently, the relatives voluntarily contacted the research centers for inclusion in this study. All the relatives received information related to this study and signed the informed consent form. Relatives who were minors were given a specific information sheet approved by the Ethics Committee, with the parents signing the consent form on their behalf.

### 2.2. Lp(a) Determinations

The blood determinations of Lp(a) were carried out by the different clinical analysis laboratories at each center for both the index cases and the relatives. In all centers, the determination of Lp(a) of the index cases was routinely included as part of the study of patients admitted for an ACS, as well as the complete lipid profile. Lp(a) was measured on plasma with the LPA Test (Tina-quant Lipoprotein(a) Gen. 2, particle-enhanced immunoturbidimetric test). Blood samples were obtained after overnight fasting and included ApoB, total cholesterol, LDLc, high-density lipoprotein cholesterol (HDLc), triglyceride, No- HDLc, GFR (glomerular filtration rate), and HbA1(%).

### 2.3. Variables’ Definitions

Clinical antecedents of patients and relatives were recorded. The presence of cardiovascular disease was defined as previous diagnosis in medical reports as coronary heart disease (including acute coronary syndrome, revascularization, or chronic stable angina), heart failure, stroke, or peripheral arterial disease. Premature coronary heart disease was defined when onset was at an age < 55 in men or <65 in women. We also registered the main indications of the treatment. The lipid treatment included in this study refers to the previous treatment of the analytical determination, both in the case of relatives and in the case of patients with ACS; in the latter, the percentage of patients with statins and ezetimibe corresponds to the previous treatment of the cardiovascular event. According to their equivalencies, intensive statin treatment was considered as the administration of 40–80 mg atorvastatin/day or 20–40 mg rosuvastatin/day.

The triglycerides/HDLc ratio was used as a surrogate maker of LDL particle size and values > 2 were assumed to be low and dense LDL particles [11].

### 2.4. Statistical Analysis

All data collected for this study were recorded in an anonymous and online database, specifically built for this purpose. Further analyses were performed with SPSS v21 and STATA 14.3 (StataCorp. 2009. Stata Statistical Software: Release 14. College Station, TX, USA, StataCorp LP).

The qualitative variables were recorded in a frequency table. The following values were obtained for each quantitative variable: mean, median, standard deviation, minimum, and maximum. As part of this descriptive study, the corresponding family percentages with elevated Lp(a) and its corresponding confidence interval were calculated.

A bivariate analysis was calculated to determine which factors could be related to this alteration. To do this, the Chi-square or Fisher’s tests were used for the qualitative variables, and in the case of the quantitative variables, their normality was studied using the Kolmogorov–Smirnov test. To study the relationship between a quantitative and a qualitative variable with two modalities, the Student’s *t*-test for independent samples or the non-parametric Mann–Whitney U test was used. In the event that the qualitative variable presented 3 or more modalities, an ANOVA or the Kruskal–Wallis non-parametric test was performed for the analysis. To determine significant differences in the results, the corresponding multiple comparisons were studied. Correlations were assessed by linear regressions. Linear regressions were also used to assess colinearity between LDLc and ApoB. For all the analyses, a value of *p* = 0.05 was considered significant.

## 3. Results

**Figure 1:**
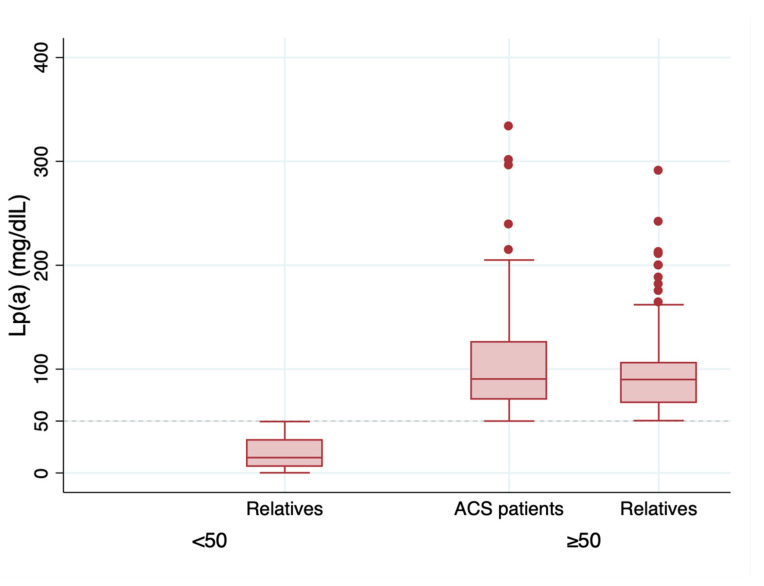
Serum levels of Lp(a) in index patients and their relatives.

A total of 413 patients were included: 180 (43.6%) were index cases and 233 were relatives (56.4%); 1.29 relatives were studied for each index case. The clinical characteristics of the cohort and each group are presented in Table 1. Mean Lp(a) levels were 82.1 mg/dL, and it was significantly lower in relatives; nonetheless, 59.4% of the relatives had Lp(a) levels ≥ 50 mg/dL and 10.8% had Lp(a) levels ≥ 120 mg/dL. As shown in Figure 1, relatives with elevated Lp(a) (≥50 mg/dL) had similar Lp(a) values as their index case (96.8 vs. 103.8 mg/dL; *p* = 0.16). As shown in Table 2, among the group of relatives, no differences in age, presence of cardiovascular risk factors, or lipid levels were observed regarding Lp(a)> or <50 mg/dL.

**Figure 2:**
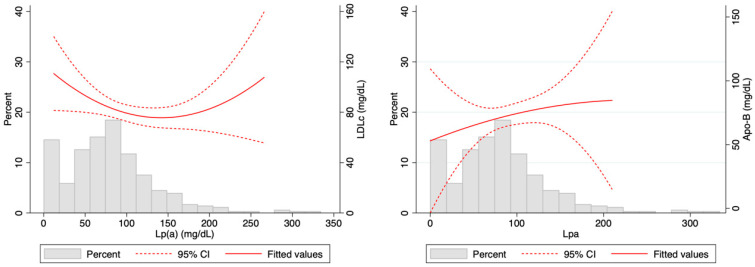
Distribution of Lp(a) and its correlation with low-density lipoprotein cholesterol (LDLc) levels (**left**) and ApoB (**right**).

**Figure 3:**
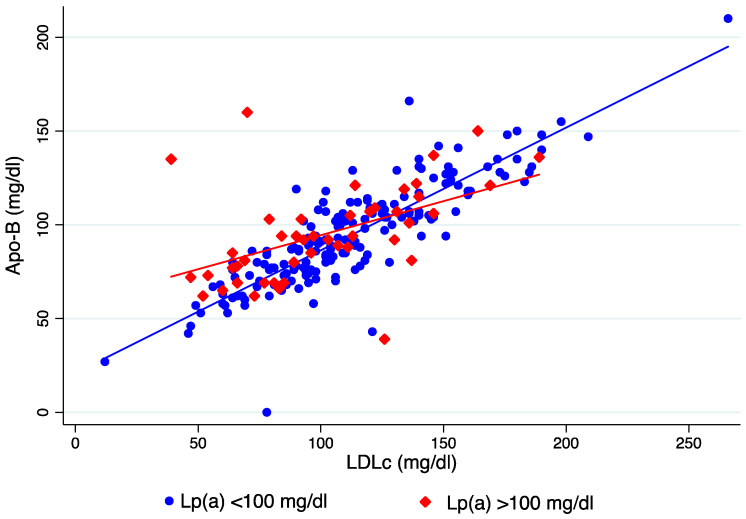
Correlation between LDLc and ApoB in patients with Lp(a) levels< or >100 mg/dL.

As expected, the correlation between LDLc and ApoB was good (β-coefficient = 0.79; *p* < 0.001), but a significant interaction of Lp(a) levels was observed in such a correlation. As depicted in Figure 2, LDLc levels had a “U-shaped” distribution according to Lp(a) as they tended to decrease if Lp(a) was >50 mg/dL, but it increased, again when Lp(a) was extremely high. In contrast, ApoB levels increased as Lp(a) increased. These results were verified as a significant correlation (*p* = 0.01) was detected for Lp(a) and LDLc levels. The correlation between LDLc and ApoB differed according to Lp(a) levels (Figure 3); in patients with Lp(a) < 100 mg/dL, β-coefficient = 0.86 (*p* < 0.001), but in patients with Lp(a) > 100 mg/dL, it was much weaker (β-coefficient = 0.54; *p* < 0.01).

## 4. Discussion

The results of this study show four relevant conclusions: (1) three out of five first-degree relatives of patients with elevated Lp(a) and an ACS also have elevated Lp(a); (2) relatives with elevated Lp(a) have similar levels as their family members that had an ACS; (3) Lp(a) levels altered the relationship between LDLc and ApoB levels; and (4) the interaction of Lp(a) modified the correlation between LDLc and ApoB levels, especially when Lp(a) was >100 mg/dL (graphical abstract). The clinical characteristics of our population are similar to previous reports [4,9], and, therefore, we believe that our results might be representative and clinically useful.

The prevalence of elevated Lp(a) in relatives was slightly higher than in other studies. In a population-based study of familial cascade for familial hypercholesterolemia (FH), which included the determination of Lp(a), they observed that 41% of the 162 family members included in the study presented elevated Lp(a) levels [10]. In the Spanish FH cohort (SAFEHEART) [12], 1 out of 2.4 relatives were identified when FH and elevated Lp(a) coexisted in the index case. If we take into account that the inheritance of the LPA [13] gene is autosomal dominant, these results are expected in terms of the proportion of relatives with elevated Lp(a), but our study provides information on a cohort of relatives with coronary disease that was not previously described. Moreover, relatives with elevated Lp(a) had similar values to the ACS patients, revealing great potential for the prevention of premature cardiovascular disease in these patients.

When we analyzed the cohort of relatives of our study, we observed a younger population with a lower presence of cardiovascular risk factors and less metabolic dysfunction, measured by glycosylated hemoglobin levels and atherogenic indices. This may be due to the fact that most of the relatives studied were children of the index cases and because there might be a time gap for the development of other risk factors and cardiovascular disease. Nonetheless, relatives with elevated Lp(a) had similar levels as the index cases, with the same risk factors as relatives with non-elevated Lp(a). These findings support the benefit of routine clinical family cascade screening [12].

According to clinical guidelines, the determination of Lp(a) could be considered in people with a family history of early cardiovascular disease or to reclassify cardiovascular risk into moderate and high risk [14,15]. Nonetheless, the 2022 consensus of the European Society of Atherosclerosis recommended its determination as once-in-a-lifetime for the assessment of cardiovascular risk [14]. Lp(a) is scarcely measured in clinical practice [15]. In a recent registry with more than four million patients, Lp(a) was determined only in 0.25% of patients in 2015 and 0.34% in 2018 [16]; the results translated into more intensive preventive measures that led to a positive effect in reducing morbidity and increasing survival.

Our study yields novel data, which could lead researchers to rethink the need to measure Lp(a) levels in the relatives of patients with coronary heart disease, due to the high possibility that they may also have it, and its implication in the increase in cardiovascular risk in this population. Although it is true that there are currently no specific treatments for the reduction of Lp(a) and there are several molecules that have been shown to cause >85% reduction in Lp(a) [17,18], if we know the excess risk in this population, we can address global cardiovascular risk earlier.

One of the strengths of our study was that we could assess the effect of Lp(a) on the correlation between LDLc and ApoB in a wide range of Lp(a) values.

The results of this registry suggest that a large percentage of relatives of ACS patients with elevated Lp(a) could be candidates for therapies that efficiently reduce Lp(a). Such therapies are currently being evaluated in randomized clinical trials, but PCSK9 inhibitors reduce Lp(a) by 25%, and subanalysis of the FOURIER trial [5] and the ODYSSEY Outcomes trial [6] demonstrated that patients with very high levels of Lp(a) obtained a significant benefit from the reduction in Lp(a) by these therapies. Nonetheless, the ongoing trials with a non-sense oligonucleotide, pelacarsen [17], or a small RNA silencer, olpasiran [18], would provide conclusive conclusions related to the reduction of Lp(a) to <50 mg/dL in patients with established IHD and elevated Lp(a) levels [19]. Although we cannot currently use therapies aimed at reducing Lp(a) in family members who have high Lp(a), since plasmapheresis is only indicated in patients with progressive cardiovascular disease and very high Lp(a), we can address the rest of the risk factors and reduce LDL levels, achieving the appropriate objectives based on their excess cardiovascular risk, as indicated by experts [14].

### Limitations

Our study has some limitations that should be addressed. First, it was an observational and cross-sectional study that can only describe the association between variables. Second, the participation of the relatives was completely free, and thereafter, a selection bias could be present. Third, results and conclusions are based on serum lipid values; genetic determinations could have provided more detailed results, and this is already planned. We believe that these limitations do not undermine the significance the results, which might be representative of clinical practice.

## 5. Conclusions

Approximately three out of five relatives of patients who have been discharged after an ACS with elevated Lp(a) also have Lp(a) > 50 mg/dL. The Lp(a) levels of relatives who have elevated Lp(a) are similar to those of family members with ACS, and its measurement is an opportunity for the assessment of cardiovascular risk and possible intervention.

## Figures and Tables

**Table 1:** Clinical characteristics of the two cohorts.

	Total	ACS Patients	Relatives	*p*
N	413	180	233	
Age	47.3 (1–89)	59.1 (35–89)	37.5 (1–89)	<0.001
Women (%)	40.4	23.9	53.2	<0.001
Hypertension (%)	30.3	54.7	12	<0.001
Diabetes Mellitus (%)	16.2	30.7	5.3	<0.001
Current smokers (%)	24.9	42.2	10.6	<0.001
Dislipidemia (%)	37	61.7	18.7	<0.001
Previous CHD (%)	2.4	22.3	4	<0.001
Peripheral arterial disease (%)	4.1	7.8	1.3	<0.001
Stroke (%)	2.4	4.5	0.9	<0.001
Thromboembolic disease (%)	1.5	2.8	0.4	<0.001
Valvulopathy (%)	3.4	6.7	0.9	<0.001
Family history				
CHD FA (%)	73.4	45.9	100	<0.001
Premature CHD FA (%)	57.5	56.6	57.8	NS
Dislipidemia FA (%)	4.6	4.7	4.9	NS
Lipid-lowering therapies				
Statins (%)	27.4	48	12.1	<0.001
Ezetimibe (%)	5.1	20.1	3.9	<0.001
Biochemical determinations				
Lp(a) (mg/dL)	82.1 (0.2–334)	103.4 (39–334)	64.9 (0.2–291)	<0.001
Total cholesterol (mg/dL)	178.8 (68–298)	172.3(68–293)	184.1 (80–298)	<0.001
HDLc (mg/dL)	49.1 (21–96)	41.2 (21–85)	55.5 (24–96)	<0.001
LDLc (mg/dL)	107.6 (12–266)	103.3(22–266)	110.9 (12–259)	0.05
Triglycerides (mg/dL)	127 (38–626)	150 (64–626)	108.5 (38–388)	<0.001
ApoB (mg/dL)	93.5 (27–210)	101.8 (30–210)	91.2 (27–150)	0.01
Non-HDL cholesterol (mg/dL)	129.9 (32–258)	131.6 (35–258)	128.6 (32–240)	NS
TC/HDLc	3.9 (1.67–8.9)	4.4 (1.7–8.9)	3.5 (1.67–8.57)	<0.001
Tg/HDLc	3 (0.52–16.91)	4 (0.96–16.92)	2.2 (0.52–12.1)	<0.001
LDLc/ApoB	1.1 (0.29–3.23)	1.1 (0.29–3.23)	1.1 (0.4–1.6)	NS
GFR (mL/min/1.72 m^2^)	94.6 (5.03–224)	83.5 (5.03–117.5)	103.6(14.8–224)	<0.001
HbA1c (%)	5.7 (2–12.5)	6.1 (2.5–12.5)	5.5 (4.4–9)	<0.001

CHD: coronary heart disease; FA: familial antecedents: Lp(a): lipoprotein(a); GFR: glomerular filtration rate; HDLc: high-density lipoprotein cholesterol; LDLc: low-density lipoprotein cholesterol; TC/HDLc: total cholesterol to HDLc ratio; and Tg/HDLc: triglycerides to HDLc ratio. HbA1c: glycosylated hemoglobin; NS: no significant.

**Table 2:** Characteristics of the group of relatives according to the presence or absence of elevated Lp(a).

	Lp(a) mg/dL		*p*
	<50	≥50	
N	94	139	
Edad	36.2	38.5	NS
Women (%)	52.2	55.3	NS
Hypertension (%)	13.0	11.5	NS
Diabetes Mellitus (%)	5.5	5.3	NS
Current smokers (%)	7.02	12.6	NS
Dislipidemia (%)	14.4	21.5	NS
FA of premature CHD (%)	60	55.8	NS
FA of dislipidemia (%)	4.4	5.3	NS
Lp(a) (mg/dL)	18.8	96.8	<0.001
Total cholesterol (mg/dL)	178.6	188.1	NS
HDLc (mg/dL)	54.3	56.3	NS
LDLc (mg/dL)	105.2	115.1	0.05
Triglycerides (mg/dL)	117.6	102.3	NS
ApoB (mg/dL)	87.1	94.2	NS
Non-HDL cholesterol (mg/dL)	124.2	131.8	NS
TC/HDLc	3.4	3.5	NS
TG/HDLc	2.5	2.03	NS
LDLc/ApoB	1.1	1.1	NS
GFR (mL/min/1.72 m^2^)	107.5	100.6	NS
HbA1c (%)	5.5	5.5	NS

Lp(a): lipoprotein(a); GFR: glomerular filtration rate; HDLc: high-density lipoprotein cholesterol; LDLc: low-density lipoprotein cholesterol; TC/HDLc: total cholesterol to HDLc ratio; and TG/HDLc: triglycerides to HDLc ratio. HbA1c: glycosylated hemoglobin; NS: no significant.

## Data Availability

The data presented in this study are available on request from the corresponding author.
